# Supplementary material for: A Comparison of Meeting Physical Activity and Screen Time Recommendations between Canadian Youth Living in Rural and Urban Communities: A Nationally Representative Cross-Sectional Analysis
Source: Int J Environ Res Public Health. 2022 Apr 6;19(7):4394. doi: 10.3390/ijerph19074394 (PMC8998429; doi:10.3390/ijerph19074394)
Supplement: Supplementary file 1 [file ijerph-19-04394-s001.zip › ijerph-1631705-supplementary.pdf]

International Journal of
Environmental Research and Public Health

MDPI

Article

A Comparison of Meeting Physical Activity and Screen Time Recommendations between Canadian Youth Living in Rural and Urban Communities: A Nationally Representative Cross-Sectional Analysis

Taru Manyanga [1,2,*], Chelsea Pelletier [3], Stephanie A. Prince [4,5], Eun-Young Lee [6], Larine Sluggett [7] and Justin J. Lang [4,8]

1 Division of Medical Sciences, University of Northern British Columbia, Prince George, BC V2N 4Z9, Canada
2 Department of Physical Therapy, Faculty of Medicine, University of British Columbia, Vancouver, BC V6T 1Z3, Canada
3 Faculty of Human and Health Sciences, School of Health Sciences, University of Northern British Columbia, Prince George, BC V2N 4Z9, Canada; chelsea.pelletier@unbc.ca
4 Centre for Surveillance and Applied Research, Public Health Agency of Canada, Ottawa, ON K1A 0K9, Canada; stephanie.prince.ware@phac-aspc.gc.ca (S.A.P.); justin.lang@phac-aspc.gc.ca (J.J.L.)
5 School of Epidemiology and Public Health, Faculty of Medicine, University of Ottawa, Ottawa, ON K1G 5Z3, Canada
6 School of Kinesiology & Health Studies, Queen's University, Kingston, ON, ON K7L 3N6, Canada; eunyoung.lee@queensu.ca
7 Northern Medical Program, Division of Medical Sciences, University of Northern British Columbia, Prince George, BC V2N 4Z9, Canada; larine.sluggett@unbc.ca
8 Department of Mathematics and Statistics, Faculty of Science, Carleton University, Ottawa, ON K1S 5B6, Canada
* Correspondence: taru.manyanga@unbc.ca

Citation: Taru, M.; Chelsea, P.; Stephanie A., P.; Eun-Young, L.; Larine, S.; Justin J., L. A Comparison of Meeting Physical Activity and Screen Time Recommendations between Canadian Youth Living in Rural and Urban Communities: A Nationally Representative Cross-Sectional Analysis. *Int. J. Environ. Res. Public Health* **2022**, *19*, 4394. https://doi.org/10.3390/ijerph19074394

Academic Editor: Paul B. Tchounwou

Received: 23 February 2022
Accepted: 3 April 2022
Published: 6 April 2022

Publisher's Note: MDPI stays neutral with regard to jurisdictional claims in published maps and institutional affiliations.

Copyright: © 2022 by the authors. Submitted for possible open access publication under the terms and conditions of the Creative Commons Attribution (CC BY) license (https://creativecommons.org/licenses/by/4.0/).

Table S1. STROBE Statement—Checklist of items that should be included in reports of cross-sectional studies.

	Item No	Recommendation
Title and abstract	1	(a) Indicate the study's design with a commonly used term in the title or the abstract: (Page 1: Title, and Abstract line 26)
		(b) Provide in the abstract an informative and balanced summary of what was done and what was found: (Page 1: Abstract lines 26–38)
		**Introduction**
Background/rationale	2	Explain the scientific background and rationale for the investigation being reported: (Page 2: lines 47–96)
Objectives	3	State specific objectives, including any pre-specified hypotheses: (Page 1 lines 24–26 and lines 93–96)
		Methods
Study design	4	Present key elements of study design early in the paper (Page 1: abstract lines 26–28 and page 3 lines 99–103)
Setting	5	Describe the setting, locations, and relevant dates, including periods of recruitment, exposure, follow-up, and data collection (Page 3: lines 99–110)
Participants	6	(a) Give the eligibility criteria, and the sources and methods of selection of participants (Page 3: lines 112–122)
Variables	7	Clearly define all outcomes, exposures, predictors, potential confounders, and effect modifiers. Give diagnostic criteria, if applicable (Page 3: lines 124–165)
Data sources/ measurement	8 *	For each variable of interest, give sources of data and details of methods of assessment (measurement). Describe comparability of assessment methods if there is more than one group (Page 3: lines 99 -110)
Bias	9	Describe any efforts to address potential sources of bias (Page 3: lines 112–122 and Table S2)
Study size	10	Explain how the study size was arrived at (Page 3: lines 112–122)
Quantitative variables	11	Explain how quantitative variables were handled in the analyses. If applicable, describe which groupings were chosen and why (Page 3–4 lines 124–186)
Statistical methods	12	(a) Describe all statistical methods, including those used to control for confounding (Page 4 lines 167–186)
		(b) Describe any methods used to examine subgroups and interactions (Page 4: lines 175–180)
		(c) Explain how missing data were addressed (Page3: lines 112–122 and Table S2)
		(d) If applicable, describe analytical methods taking account of sampling strategy (Page 4: lines 167–172)
		(e) Describe any sensitivity analyses (Table S2)
		**Results**
Participants	13 *	(a) Report numbers of individuals at each stage of study—e.g., numbers potentially eligible, examined for eligibility, confirmed eligible, included in the study, completing follow-up, and analysed (Page 4: lines 188–202)
		(b) Give reasons for non-participation at each stage (n/a)
		(c) Consider use of a flow diagram (n/a)
Descriptive data	14 *	(a) Give characteristics of study participants (e.g., demographic, clinical, social) and information on exposures and potential confounders (Table 1)
		(b) Indicate number of participants with missing data for each variable of interest (Page 3: lines 112–122 and Table S2)
Outcome data	15 *	Report numbers of outcome events or summary measures (n/a)
Main results	16	(a) Give unadjusted estimates and, if applicable, confounder-adjusted estimates and their precision (e.g., 95% confidence interval). Make clear which confounders were adjusted for and why they were included (Page 4–11; tables 1–4)
		(b) Report category boundaries when continuous variables were categorized (Page 3–4: lines 125–165)
		(c) If relevant, consider translating estimates of relative risk into absolute risk for a meaningful time period (n/a)
Other analyses	17	Report other analyses done—e.g., analyses of subgroups and interactions, and sensitivity analyses (n/a)
		**Discussion**

Key results	18	Summarise key results with reference to study objectives (Page 9: lines 238–253)
Limitations	19	Discuss limitations of the study, taking into account sources of potential bias or imprecision. Discuss both direction and magnitude of any potential bias (Page 10–11: lines 328–343)
Interpretation	20	Give a cautious overall interpretation of results considering objectives, limitations, multiplicity of analyses, results from similar studies, and other relevant evidence (Page 11: lines 350–354)
Generalisability	21	Discuss the generalisability (external validity) of the study results (Page 1: lines 26–28 and page 11 line 328)
**Other Information**		
Funding	22	Give the source of funding and the role of the funders for the present study and, if applicable, for the original study on which the present article is based (Page 11; line 363)

* Give information separately for exposed and unexposed groups. Note: An Explanation and Elaboration article discusses each checklist item and gives methodological background and published examples of transparent reporting. The STROBE checklist is best used in conjunction with this article (freely available on the Web sites of PLoS Medicine at http://www.plosmedicine.org/, Annals of Internal Medicine at http://www.annals.org/, and Epidemiology at http://www.epidem.com/). Information on the STROBE Initiative is available at www.strobe-statement.org.

Table S2. Respondents with complete versus missing physical activity and screen time data.

**Variable**	**Missing (*N* = 1786)**	**Complete (*N* = 6176)**
	**% (95%CI)**	**% (95%CI)**
Residence		
Urban	81.4 (79.1, 83.6)	81.2 (80.1, 82.2)
Age group		
12–13 years	45.7 (42.0, 49.5)	31.3 (29.7, 33.0) *
14–15 years	33.6 (30.1, 37.1)	33.8 (32.1, 35.4)
16–17 years	20.7 (17.8, 23.7)	34.9 (33.2, 36.6) *
BMI category		
Thinness	4.4 (2.5, 6.2)	2.5 (1.9, 3.1)
Normal weight	69.6 (65.9, 73.3)	72.3 (70.7, 74.0)
Overweight	18.2 (15.0, 21.4)	17.3 (15.9, 18.7)
Obese	7.8 (6.0, 9.6)	7.9 (6.9, 8.8)
Sex		
Female	48.3 (45.1, 51.5)	48.8 (47.8, 49.7)
Perceived general health		
Very good/excellent	70.6 (67.3, 73.8)	75.7 (74.2, 77.2) *
Perceived mental health		
Very good/excellent	72.6 (69.2, 75.9)	75.5 (73.9, 77.2)

* Respondents with complete data are significantly different from those with missing data at $p < 0.05$; BMI = body mass index; Meeting physical activity recommendation = an average of 60 min per day of total moderate-to-vigorous intensity PA; meeting screen time recommendation ≤ 2 h per day of recreational screen time.
